# Plasma osteopontin as a biomarker of Alzheimer’s disease and vascular cognitive impairment

**DOI:** 10.1038/s41598-021-83601-6

**Published:** 2021-02-17

**Authors:** Yuek Ling Chai, Joyce R. Chong, Ainiah R. Raquib, Xin Xu, Saima Hilal, Narayanaswamy Venketasubramanian, Boon Yeow Tan, Alan P. Kumar, Gautam Sethi, Christopher P. Chen, Mitchell K. P. Lai

**Affiliations:** 1 Department of Pharmacology, Yong Loo Lin School of Medicine, National University of Singapore, Unit 09-01, Centre for Translational Medicine (MD6), 14 Medical Drive, Singapore, 117599 Singapore; 2 Memory Aging and Cognition Centre, National University Health System, Singapore, Singapore; 3 Saw Swee Hock School of Public Health, National University of Singapore, Singapore, Singapore; 4 St. Luke’s Hospital, Singapore, Singapore; 5 Raffles Neuroscience Centre, Raffles Hospital, Singapore, Singapore; 6 Cancer Science Institute of Singapore and Department of Pharmacology, Yong Loo Lin School of Medicine, National University of Singapore, Singapore, Singapore

**Keywords:** Neurological disorders, Biomarkers

## Abstract

Cerebrovascular disease (CeVD) and neurodegenerative dementia such as Alzheimer’s disease (AD) are frequently associated comorbidities in the elderly, sharing common risk factors and pathophysiological mechanisms including neuroinflammation. Osteopontin (OPN) is an inflammatory marker found upregulated in vascular diseases as well as in AD. However, its involvement in vascular dementia (VaD) and pre-dementia stages, namely cognitive impairment no dementia (CIND), both of which fall under the spectrum of vascular cognitive impairment (VCI), has yet to be examined. Its correlations with inflammatory cytokines in cognitive impairment also await investigation. 80 subjects with no cognitive impairment (NCI), 160 with CIND and 144 with dementia were included in a cross-sectional study on a Singapore-based memory clinic cohort. All subjects underwent comprehensive clinical, neuropsychological and brain neuroimaging assessments, together with clinical diagnoses based on established criteria. Blood samples were collected and OPN as well as inflammatory cytokines interleukin (IL)-6, IL-8 and tumor necrosis factor (TNF) were measured using immunoassays. Multivariate regression analyses showed significant associations between increased OPN and VCI groups, namely CIND with CeVD, AD with CeVD and VaD. Interestingly, higher OPN was also significantly associated with AD even in the absence of CeVD. We further showed that increased OPN significantly associated with neuroimaging markers of CeVD and neurodegeneration, including cortical infarcts, lacunes, white matter hyperintensities and brain atrophy. OPN also correlated with elevated levels of IL-6, IL-8 and TNF. Our findings suggest that OPN may play a role in both VCI and neurodegenerative dementias. Further longitudinal analyses are needed to assess the prognostic utility of OPN in disease prediction and monitoring.

## Background

Over 50 million people worldwide are affected by dementia, and the number is estimated to almost triple by the year 2050 (World Alzheimer Report 2018). While dementia is an umbrella term for symptoms associated with memory decline and deterioration in one’s ability to perform activities of daily living, there are various etiologies underlying dementia, of which Alzheimer’s disease (AD) and vascular dementia (VaD) are the top two commonest forms. AD is a progressive neurodegenerative disease characterized by cortical deposition of amyloid plaques and neurofibrillary tangles, whereas VaD is generally associated with small vessel cerebrovascular diseases (CeVD), and falls under the spectrum of vascular cognitive impairment (VCI). Nevertheless, AD and VaD are often found concomitantly as mixed dementia, and also share comorbidities and overlapping pathophysiological mechanisms. Neuroinflammation, for instance, has been observed in both AD and VaD. Studies have reported the co-localization of activated microglia and astrocytes with amyloid plaques in AD brains^1^, together with elevated levels of various pro-inflammatory cytokines in blood and cerebrospinal fluid^2^. Similarly, activated glial cells around vascular lesions in postmortem brains^3,4^ as well as increased cytokines and chemokines have been reported in blood and cerebrospinal fluid of VaD patients^5–8^. Furthermore, vascular risk factors are known to contribute to AD progression^9^. In particular, stroke, cardiovascular disease, hypertension and atherosclerosis are known risk factors of cognitive impairment and AD^10–12^. It has been speculated that neuroinflammation may again play a part, as deleterious inflammatory responses at the site of injury are triggered by vascular insults as well as the resultant compromise of endothelial and blood–brain barrier functions^13,14^. Therefore, in ongoing efforts to discover and develop blood-based biomarkers of VCI and AD^15–19^, regulators of inflammation may represent attractive targets. Of these, osteopontin (OPN) has been shown to play an important role in the pathophysiology of CeVD and inflammation.

OPN, also known as early T-lymphocyte activation or secreted phosphoprotein 1, is an extracellular phosphoprotein expressed in various tissues and cells in response to stress and injury. It may be upregulated under hypoxic conditions^20,21^, atherosclerosis^22,23^, angioplasty^24^, cerebral ischemia^25,26^, and by pro-inflammatory cytokines such as interleukin-1β and interleukin-6^27,28^. OPN has in turn been shown to regulate macrophage infiltration and cytokine production^29–31^. In the past decade, accumulating evidence pointed to the involvement of OPN in inflammation-associated neurological disease. In AD, higher OPN levels have been reported in the brain^32^ as well as in CSF^33^. Elevation of OPN was also observed in the plasma of patients with AD of less than 2 years, but not in patients with longer disease duration^34^. However, whilst studies have focused on AD, little is known about the status of OPN in VaD. The putative link with pre-dementia stages of VCI, i.e., cognitive impairment no dementia (CIND) also awaits investigation. In the present study, we aimed to examine the associations of blood OPN with AD and VCI, as well as with their neuroimaging and neurocognitive features. Given the role of OPN as a central regulator during inflammatory processes especially in regulating cytokine production, its potential associations with circulating inflammatory cytokine levels were also assessed.

## Methods

### Study cohort

The present case–control study consisted of subjects with CIND and dementia recruited from two study sites in Singapore (National University Hospital and Saint Luke’s Hospital). Cognitively normal controls (No Cognitive Impairment, NCI), defined as cognitively normal on objective neuropsychological assessments, were recruited from both memory clinics and the community^35^. Institutional Review Board approval for the study, which was conducted in accordance with the Declaration of Helsinki, was obtained from the Singapore National Healthcare Group Domain-Specific Review Board (reference 2010/00017; study protocol DEM4233). Written informed consent was obtained for all participants or their caregivers in their preferred language prior to study recruitment.

### Examination procedures

All subjects underwent standard physical, clinical, blood tests and neuropsychological assessments as well as neuroimaging scans at the National University of Singapore. Detailed study procedures have been described previously^35^.

#### Blood biomarkers measurements

Non-fasting blood was drawn from study participants into both serum-separating tubes (SST) and ethylenediaminetetraacetic acid (EDTA) tubes, and processed by centrifugation at 2000*g* for 10 min at 4 °C to derive serum and plasma, respectively, which were extracted, aliquoted into Eppendorf tubes and stored at − 80 °C until use. All blood-based biomarkers were measured in duplicates and analyzed blinded to subject characteristics and clinical status.

OPN concentrations were measured by quantitative sandwich immunoassays (Quantikine catalogue number SOST00, R&D Systems Inc., Minneapolis, MN, USA) in accordance to manufacturer’s instructions. Briefly, stored plasma samples were thawed, diluted 25-fold in calibrator diluent buffer before adding to antibody-coated plates. Stabilized chromogen tetramethylbenzidine was then added into the wells at the recommended volumes, and color development was stopped after 30 min by the addition of the provided stop solution. Absorbance was measured at 450 nm on a microplate reader (BioTek, Winooski, VT, USA) with background subtraction at 570 nm. A standard curve ranging from 0.313 to 20 ng/mL was generated for each assay and fitted to a 4-parameter logistic model with weighted R-squared correlation coefficient > 0.99. Sample concentrations read from the standard curve were multiplied by the dilution factor of 25 to obtain the actual OPN levels in plasma.

Inflammatory cytokines, namely IL-6, IL-8 and TNF, were measured using multiplex xMAP-based Luminex immunoassays (MILLIPLEX, catalogue number HADK2MAG-61K, Merck Millipore, Billerica, MA, USA), as described previously^36^. Briefly, SST serum samples were incubated overnight with a mixture of fluorescent-coded magnetic beads coated with specific capture antibody against each cytokine. This was followed by the addition of biotinylated antibody and streptavidin–phycoerythrin conjugates. The median fluorescent intensity (MFI) were measured on a Luminex 200 machine with xPONENT software. The standard curves were fitted to a 5-parameter logistic model, ranging from 0.96 to 15,000 pg/mL for IL-6, and 0.64–10,000 pg/mL for both IL-8 and TNF.

#### Neuropsychological assessments

Cognitive tests, which included the Mini-Mental State Examination (MMSE), the Montreal Cognitive Assessment (MoCA) and a locally validated, detailed neuropsychological test battery^37^, were administered to all subjects by trained research psychologists. The test battery assessed seven cognitive domains, including Executive Function, Attention, Language, Visuomotor Speed, Visuoconstruction, Visual Memory and Verbal Memory (see Additional File: Supplementary Table 1 for a summary of the component tests). Raw scores for all individual tests on the test battery were transformed to standardized z-scores using the mean values and standard deviations (SDs) of the study reference group (NCI). The z-score for each domain was then derived by averaging the z-scores of individual tests and standardized using the means and SDs of the reference group. To obtain the global cognitive z-score for each subject, the domain z-scores were averaged and standardized using the means and SDs of the reference group.

#### Neuroimaging

Magnetic resonance imaging (MRI) scans were performed on a 3-T Siemens Magnetom Trio Tim scanner, using a 32-channel head coil, at the Clinical Imaging Research Centre, National University of Singapore. Subjects with claustrophobia, contraindications for MRI, or those who were unable to tolerate the procedure were excluded. All MRIs were graded by one radiologist and two clinicians blinded to the neuropsychological and clinical data. The sequences included T1-weighted Magnetization Prepared Rapid Gradient Recalled Echo (MPRAGE), Fluid Attenuated Inversion Recovery (FLAIR), T2-weighted and Susceptibility Weighted Imaging sequences. Presence of lacunes and cortical infarcts were defined on FLAIR and T2 sequences using STRIVE criteria^38^, whereas white matter hyperintensities (WMH) were graded using the Age-Related White Matter Changes scale (ARWMC)^39^. Significant CeVD was defined as the presence of cortical strokes and/or ≥ 2 lacunes and/or confluent WMH (ARWMC score ≥ 8) in two regions of the brain, as described previously^35^.

Brain atrophy as a neuroimaging marker for neurodegeneration was also assessed on the MRI scans, with the degree of central and cortical atrophy assessed by ventricular or subarachnoid space and sulcal dilation on axial sections and rated using a 4-point scale (0—normal, 1—mild, 2—moderate or 3—severe)^40^. Additionally, medial temporal lobe atrophy was defined by the widening of the choroid fissure, widening of temporal horn and loss of hippocampal height as seen on coronal sections, and was graded using the 5-point Scheltens’ scale (0—normal, 1—mild, 2—mild–moderate, 3—moderate, 4—severe)^41^. The presence of significant cortical, central and medial temporal atrophy was defined by a score of ≥ 2 on the respective scales, as previously described^42^.

#### Clinical diagnoses of cognitive impairment and dementia

Diagnoses of cognitive impairment and dementia were made at weekly consensus meetings by study clinicians and neuropsychologists. CIND was determined by clinical judgment based on published guidelines^43^, namely, impairment in at least one domain of the neuropsychological test battery without any significant dysfunction in activities of daily living. Participants were considered to have failed a test if they scored 1.5 standard deviation (SD) below education-adjusted cut-off values on each individual test. Failure in at least half of the tests in each domain was considered as impairment in that domain. The diagnosis of dementia was based on the diagnostic and statistical manual of mental disorders, 4th edition (DSM-IV) criteria. The etiology of dementia was further classified, with AD being diagnosed based on the National Institute of Neurological and Communicative Disorders and Stroke and the Alzheimer's Disease and Related Disorders Association (NINCDS-ADRDA) criteria^44^, while vascular dementia (VaD) being diagnosed using the National Institute of Neurological Disorders and Stroke-Association Internationale pour la Recherché et l' Enseignement en Neuroscience (NINDS-AIRENS) criteria^45^.

#### Assessments of other risk factors

Risk factors such as hypertension, hyperlipidemia, diabetes and cardiovascular diseases were ascertained from clinical interview and medical records and classified as present or absent. Hypertension was defined as systolic blood pressure ≥ 140 mmHg and/or diastolic blood pressure ≥ 90 mmHg or use of antihypertensive medications. Diabetes mellitus was defined as glycated hemoglobin ≥ 6.5%, or on medication. Hyperlipidemia was defined as total cholesterol levels ≥ 4.14 mM, or on medication. Cardiovascular disease was classified as a previous history of atrial fibrillation, congestive heart failure and myocardial infarction. Education status was categorized as low (not exceeding primary school education) or high (beyond primary school education). Apolipoprotein E (APOE) genotyping was performed as described previously^46^, and APOE ε4 carrier status was defined as having at least one ε4 allele.

### Statistical analysis

Statistical analyses were performed using Statistics software (version 21, IBM SPSS, Chicago, IL, USA) and follows our previously published approach^36,47,48^. First, analyses of variance (ANOVA) and Chi-square tests were used to compare the characteristics of the cases and controls groups. Given that OPN was not normally distributed (Shapiro–Wilk test *p* < 0.001, skewness = 2.04, kurtosis = 5.69), OPN levels were categorized into tertiles and included as a determinant, whereas CIND and dementia were defined as outcomes. Binary logistic regression analysis with odds ratios (OR) and 95% confidence intervals (CI) were first computed for CIND and AD. Further regression analyses were performed for both CIND and AD stratified by significant CeVD defined by MRI. The models were adjusted for age, education, APOE ε4 carrier, hypertension, diabetes and heart disease as covariates, as these variables were not matched between groups (see Table 1).

**Table 1:** Baseline demographic characteristics of control and cases (n = 378).

	NCI (n = 80)	CIND (n = 158)	Dementia (n = 140)	*p*-value
Age, mean (SD), year	68.8 (6.3)	72.0 (8.2)*	75.7 (7.8)*^†^	** < 0.01**
Female, n (%)	41 (51.2)	79 (50.0)	86 (61.4)	0.11
Education ≤ elementary, n (%)	24 (30.0)	79 (50.0)*	98 (70.0)*^†^	** < 0.01**
Hypertension, n (%)	45 (56.3)	108 (68.4)	118 (84.3)*^†^	** < 0.01**
Diabetes, n (%)	18 (22.5)	54 (34.2)	62 (44.3)*	**0.01**
Cardiovascular disease, n (%)	4 (5.0)	19 (12.0)	26 (18.6)*	**0.01**
Hyperlipidemia, n (%)	54 (67.5)	120 (75.9)	109 (77.9)	0.22
APOE ε4 carrier, n (%)	14 (17.5)	47 (29.7)	45 (32.1)	0.06
Osteopontin, median (IQR), ng/ml	59.4 (22.1)	65.0 (28.4)	81.8 (42.2)*^†^	** < 0.01**

Bold values indicates *p* < 0.05 using ANOVA or Chi-square tests.

CIND, cognitive impairment no dementia; IQR, interquartile range; n, number; NCI, non-cognitive impairment; SD, standard deviation.

*Significantly different from NCI (*post-hoc* test, *p* < 0.05).

^†^Significantly different from CIND (*post-hoc* test, *p* < 0.05).

Diagnostic accuracies of OPN were assessed with logistic regression models and receiver operating characteristic (ROC) curve analyses, using R statistical software^49^ with the *pROC* package^50^. Predicted probabilities of the continuous plasma OPN values in discriminating each diagnostic class (i.e. CIND without CeVD, CIND with CeVD, AD without CeVD, AD with CeVD and VaD) from NCI or from all other diagnoses were obtained from binary logistic regression models built on the same approach described above. Predicted probabilities were then set as predictor while each diagnostic class was set of the outcome in the ROC analyses. Area under curves (AUC) and 95% CIs of each ROC analysis were computed using DeLong’s method^51^, whereas the sensitivity and specificity values were calculated at Youden index thresholds using bootstrap procedures with 2000 iterations^52^. Unadjusted models were first assessed, followed by adjustment to covariates listed above.

To examine the relationship between OPN and CeVD, as well as between OPN and brain atrophy, we performed multivariate regression analyses with log-transformed OPN included as a determinant and the specific brain MRI markers defined as outcomes. Poisson regression models with risk ratios (RR) and 95% CI were constructed for the count of cortical and lacunar infarct, linear regression models with mean differences (MD) and 95% CI were used for the ARWMC visual scores for WMH grading, and binary logistic regression with OR and 95% CI were performed for brain atrophy. All models were first adjusted for age and gender, and subsequently with additional covariates including APOE ε4 carrier, hypertension, diabetes and heart disease.

In order to explore the associations between OPN and neurocognitive performance, general linear regression analyses were performed with log-transformed OPN included as a determinant and the standardised z-scores of global as well as domain-based cognition were defined as outcomes, adjusting for age, gender, education, hypertension, diabetes and heart disease. Similarly, to examine the associations between OPN and other circulating inflammatory markers (i.e. IL-6, IL-8 and TNF), general linear regression analyses were performed with log-transformed OPN included as a determinant and the individual cytokines defined as outcomes, adjusting for age, hypertension, hyperlipidemia, diabetes and heart disease. *P* values < 0.05 were considered statistically significant.

### Ethics approval and consent to participate

Institutional Review Board approval for the study was obtained from the Singapore National Healthcare Group Domain-Specific Review Board (reference 2010/00017; study protocol DEM4233). Written informed consent was obtained for all participants in their preferred language prior to study recruitment.

### Consent for publication

All authors gave consent for publication.

## Results

### Baseline characteristics of study subjects

A total of 459 subjects were recruited from August 2010 to July 2015, of which 378 had sufficient MRI data and baseline plasma available for OPN analysis. In this study, there were 80 with (21.2%) NCI, 158 (41.8%) with CIND, and 140 (37.0%) with dementia, of whom 109 (28.8%) were AD while 31 (8.2%) were VaD. Table 1 shows the baseline demographic characteristics and plasma OPN levels of the study cohort. Cases were significantly older, had lower education and higher prevalence of hypertension, diabetes and cardiovascular disease compared to NCI.

### Associations of OPN with CIND and dementia in the presence or absence of significant CeVD

Table 1 shows that plasma OPN levels were significantly higher in dementia compared to both NCI and CIND (Kruskal–Wallis with Dunn’s *post-hoc* test, p < 0.001). After adjustment for risk factors, significant association was only observed between higher OPN and dementia (OR = 8.7; 95% CI 3.3 to 23.1), but not with CIND (OR = 2.1; 95% CI 0.9 to 4.8), as shown in Table 2. Given the possible effect of CeVD, logistic regression analyses were repeated after segregating the cognitive groups based on the subjects’ CeVD status. As seen in Table 3, higher OPN levels were significantly associated with all VCI groups, namely CIND with CeVD (OR = 3.1; 95% CI 1.1 to 8.5), AD with CeVD (OR = 5.1; 95% CI 1.5 to 16.6) and VaD (OR = 8.6; 95% CI 1.8 to 41.7). Interestingly, in the absence of significant CeVD, higher OPN levels were also significantly associated with AD (OR = 15.3; 95% CI 3.2 to 73.6), but not with CIND without CeVD (OR = 1.5; 95% CI 0.6 to 3.9).

**Table 2:** Association between osteopontin and cognitive impairment, expressed in odds ratios (OR) and 95% confidence interval (CI).

Osteopontin	CIND (n = 158) OR (95% CI)^a^	Dementia (n = 140) OR (95% CI)^a^
1st tertile	1	1
2nd tertile	0.8 (0.4–1.6)	1.5 (0.7–3.5)
3rd tertile	2.1 (0.9–4.8)	**8.7 (3.3–23.1)**

Bold values indicates *p* < 0.05 using binary logistic regression.

CIND, cognitive impairment no dementia; n, number; OR, odds ratio; CI, confidence interval.

^a^Adjusted for age, education, hypertension, diabetes and cardiovascular disease.

**Table 3:** Association between osteopontin and cognitive impairment stratified by presence of significant cerebrovascular disease (CeVD), expressed in odds ratios (OR) and 95% confidence interval (CI).

Osteopontin	Significant cerebrovascular disease (CeVD)				
	Absence		Presence		
	CIND without CeVD (n = 79) OR (95% CI)^a^	AD without CeVD (n = 40) OR (95% CI)^a^	CIND with CeVD (n = 79) OR (95% CI)^a^	AD with CeVD (n = 69) OR (95% CI)^a^	VaD (n = 31) OR (95% CI)^a,b^
1st tertile	1	1	1	1	1
2nd tertile	0.5 (0.2–1.0)	1.1 (0.2–5.9)	1.3 (0.6–3.0)	1.2 (0.4–3.6)	3.3 (0.8–41.7)
3rd tertile	1.5 (0.6–3.9)	**15.3 (3.2–73.6)**	**3.1 (1.1–8.5)**	**5.1 (1.5–16.6)**	**8.6 (1.8–41.7)**

Bold values indicates *p* < 0.05 using binary logistic regression.

Abbreviations: CeVD, cerebrovascular disease; CIND, cognitive impairment no dementia; AD, Alzheimer’s disease; VaD, vascular dementia; OR, odds ratio; CI, confidence interval.

^a^Adjusted for age, education, hypertension, diabetes and cardiovascular disease.

^b^Estimated using Firth logistic regression due to separation where hypertension perfectly predicts VaD.

**Figure 1:**
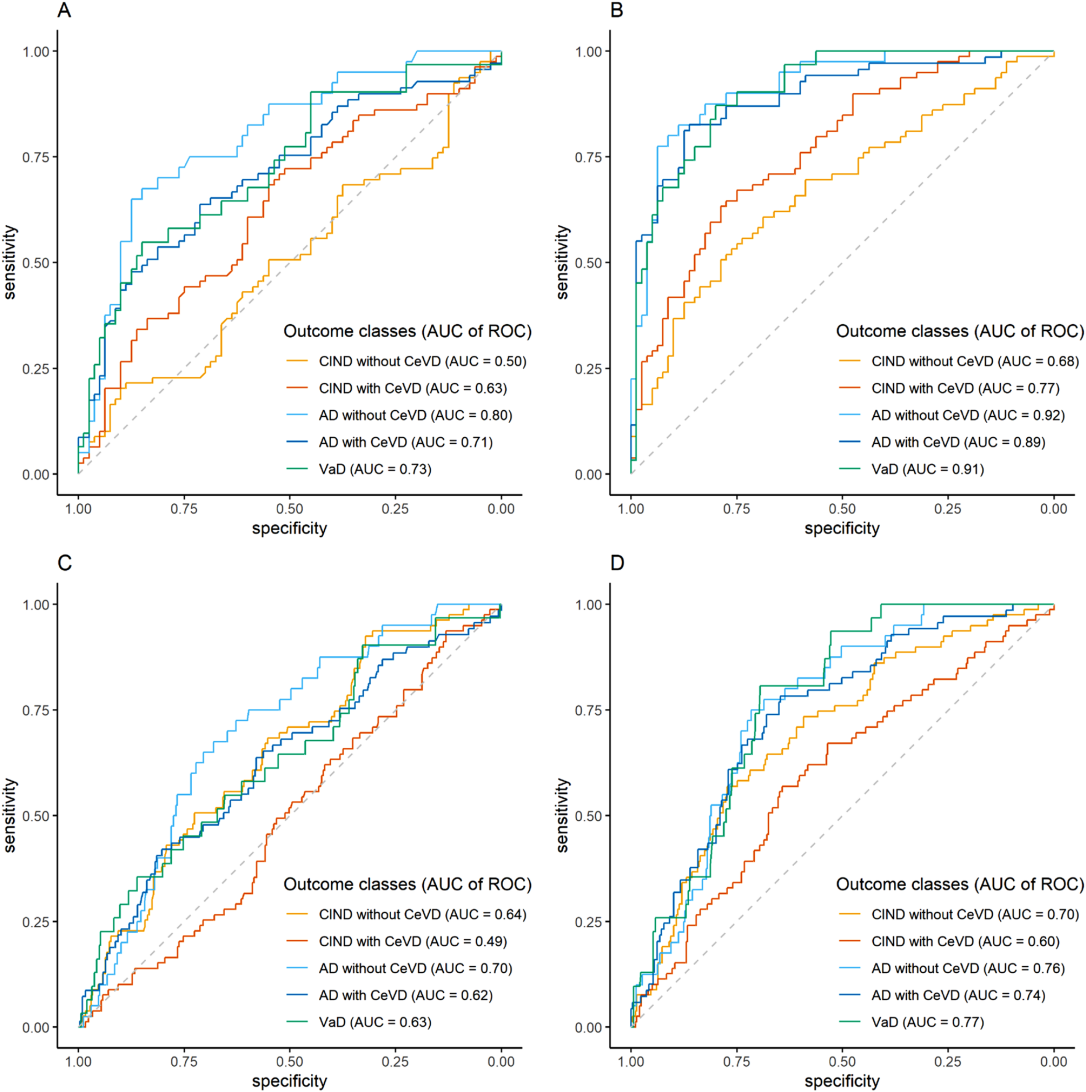
ROC curves of plasma OPN for discriminating each diagnostic outcome from (**A**,**B**) NCI or from (**C**,**D**) all other diagnoses (n = 378).

Using ROC analyses, the potential diagnostic value of plasma OPN was assessed. Additional File: Supplementary Table 2 showed that plasma OPN are reasonably good at discriminating dementia, namely AD without CeVD (AUC = 0.80, sensitivity = 72.5%, specificity = 85%), AD with CeVD (AUC = 0.71, sensitivity = 58%, specificity = 82.5%) and VaD (AUC = 0.73, sensitivity = 64.5%, specificity = 83.8%) from NCI controls. The diagnostic values of plasma OPN assessed by AUC, sensitivity and specificity values for each diagnostic class were markedly improved after adjusting for multiple covariates including age, education, APOE4 and other vascular risk factors (Additional File: Supplementary Table 2). Besides discriminating the cases from NCI, the potential diagnostic ability for plasma OPN in discriminating each diagnostic group from all other diagnoses was also assessed. As seen in Additional File: Supplementary Table 3, plasma OPN has potentially good diagnostic value after adjusting for multiple covariates, in particular for AD without CeVD (AUC = 0.78, sensitivity = 92.3%, specificity = 59.7%), AD with CeVD (AUC = 0.79, sensitivity = 81%, specificity = 69.9%) and VaD (AUC = 0.81, sensitivity = 95.7%, specificity = 63.1%). Graphical illustrations of the ROC analyses are presented in Fig. 1.

### Associations of OPN with MRI markers of CeVD and atrophy

The associations of OPN levels with MRI markers of CeVD and atrophy were assessed. Table 4 shows that every tenfold increase in OPN levels was significantly associated with increases in cortical infarct count (RR = 10.72; 95% CI 3.11 to 36.93), lacune count (RR = 4.27; 95% CI = 1.96 to 9.28) and ARWMC scores (MD = 5.21; 95% CI = 2.44 to 7.99). On the other hand, Table 5 shows that every tenfold increase in OPN levels were also associated with all three neuroimaging markers of brain atrophy, namely central atrophy (OR = 18.00; 95% CI 3.86 to 84.05), cortical atrophy (OR = 22.67; 95% CI 3.65 to 140.82) and medial temporal atrophy (OR = 55.96; 95% CI 9.51 to 329.32). All models were adjusted for age and gender, followed by other risk factors. All associations remain significant even when the analyses were restricted to the cognitively impaired groups (i.e. CIND and dementia) (Tables 4 and 5).

**Table 4:** Association between osteopontin and markers of cerebrovascular disease (CeVD), expressed in risk ratios (RR) or mean differences (MD) and the respective 95% confidence interval (CI).

	WMH by ARWMC scores MD (95% CI)	Number of cortical infarcts RR (95% CI)	Number of lacunes RR (95% CI)
**Osteopontin**^**a**^			
All subjects (max n = 378)			
Model I^b^	**5.6 (2.9–8.4)**	**4.8 (1.4–17.4)**	**3.5 (1.6–7.8)**
Model II^b^	**4.4 (1.7–7.1)**^c^	3.3 (0.9–12.7)^d^	1.5 (0.6–3.6)^e^
Cognitive impairment groups (max n = 298)			
Model I^b^	**4.6 (1.3–7.7)**	**4.2 (1.1–15.8)**	2.3 (1.0–5.1)
Model II^b^	**3.9 (0.8–6.9)**^c^	3.2 (0.8–12.7)^d^	1.2 (0.5–2.8)^e^

Bold values indicates *p* < 0.05 using Poisson or linear regression.

CeVD, cerebrovascular disease; WMH, white matter hyperintensities; ARWMC, age-related white matter changes; max n, maximum number; RR, risk ratio; MD, mean difference; CI, confidence interval.

^a^Log-transformed.

^b^Adjusted for age, gender, APOE ε4 carrier, hypertension, hyperlipidemia, cardiovascular disease, and additionally for ^c^ cortical infarct and lacunes, ^d^ WMH and lacunes, or ^e^ WMH and cortical infarct.

**Table 5:** Association between osteopontin and brain atrophy, expressed in odds ratios (OR) and 95% confidence interval (CI).

	Central atrophy OR (95% CI)^b^	Cortical atrophy OR (95% CI)^b^	Medial temporal atrophy OR (95% CI)^b^
**Osteopontin**^**a**^			
All subjects (max n = 377)	**22.2 (4.3–114.3)**	**46.4 (6.6–328.1)**	**49.3 (9.2–263.9)**
Cognitive impairment groups (max n = 297)	**17.9 (3.2–100.8)**	**18.3 (2.3–146.2)**	**26.6 (4.6–155.2)**

Bold values indicates *p* < 0.05 using binary logistic regression.

CeVD, cerebrovascular disease; WMH, white matter hyperintensities; ARWMC, age-related white matter changes; max n, maximum number; OR, odds ratio; CI, confidence interval.

^a^Log-transformed.

^b^Adjusted for age, gender, education, hypertension, diabetes and heart disease.

### Associations of OPN with neurocognitive performance

Given that neurodegeneration and CeVD contribute to cognitive impairment, the associations between OPN and neurocognitive performance were assessed. As shown in Table 6, every tenfold increase in OPN plasma levels was significantly associated with poorer global cognition (MD of z-scores = − 5.8; 95% CI − 7.7 to 3.9). Increased OPN was also significantly associated with worse neurocognitive performance on all individual domains, including the two memory domains namely verbal memory (MD = − 2.2, 95% CI − 3.2 to − 1.2) and visual memory (MD = -4.5, 95% CI − 6.3 to − 2.6) as well as the five non-memory domains, namely executive function (MD = − 3.5, 95% CI − 5.0 to 2.0), attention (MD = − 5.8, 95% CI − 8.2 to − 3.5), language (MD = − 4.4, 95% CI − 6.2 to − 2.7), visuomotor speed (MD = 95% CI − 2.7, − 3.7 to − 1.6) and visuoconstruction (MD = − 5.2, 95% CI − 6.8 to − 3.6). All regression models were adjusted for age, gender, education and other vascular risk factors (Table 6).

**Table 6:** Association of plasma osteopontin (log-transformed) with global and domain-based neurocognitive domains (in z-scores), expressed in mean difference (MD) and 95% confidence interval (CI).

	Neurocognitive performance, z-scores (n = 378) MD (95% CI)^b^	
Osteopontin^a^	Global	**− 5.8 (− 7.7, − 3.9)**
	Executive function	**− 3.5 (− 5.0, − 2.0)**
	Attention	**− 5.8 (− 8.2, − 3.5)**
	Language	**− 4.4 (− 6.2, − 2.7)**
	Visuomotor speed	**− 2.7 (− 3.7, − 1.6)**
	Visuoconstruction	**− 5.2 (− 6.8, − 3.6)**
	Verbal memory	**− 2.2 (− 3.2, − 1.2)**
	Visual memory	**− 4.5 (− 6.3, − 2.6)**

Bold values indicates p < 0.05 using linear regression.

MD, mean difference; CI, confidence interval.

^a^Log-transformed.

^b^Adjusted for age, gender, education, hypertension, diabetes and cardiovascular disease.

### Associations of OPN with inflammatory markers

General linear regression analyses were performed to examine the associations between OPN and cytokines. Out of the 378 subjects with OPN measurements available, cytokine measurement was not performed for one NCI and one AD subjects due to insufficient blood sample available, resulting in a total of 376 subjects with all four blood biomarkers analyzed. For cases whose IL-6 concentrations fell below detectable range (27 cases in NCI group, 50 cases in CIND group, 20 cases in AD group and 8 in VaD group), the lowest detectable value of 0.2 pg/mL was used in statistical analyses. Table 7 shows that every 1% increase in OPN was significantly associated with an increase in each of the three cytokines (in percentage), namely IL-6 (MD = 0.87; 95% CI 0.50 to 1.25), IL-8 (MD = 0.30; 95% CI 0.17 to 0.44) and TNF (MD = 0.31; 95% CI 0.15 to 0.48). All regression models were adjusted for age and vascular risk factors, and all associations remained significant even when confined to the cognitive impairment groups only (Table 7).

**Table 7:** Associations between osteopontin and inflammatory markers, expressed in mean difference (MD) and 95% confidence interval (CI).

	IL-6^a^ MD (95% CI)^b^	IL-8^a^ MD (95% CI)^b^	TNF^a^ MD (95% CI)^b^
**Osteopontin**^**a**^			
All subjects (max n = 376)	**0.9 (0.5–1.2)**	**0.3 (0.1–0.4)**	**0.3 (0.1–0.5)**
Cognitive impairment (max n = 297)	**0.9 (0.5–1.3)**	**0.3 (0.1–0.5)**	**0.3 (0.1–0.4)**

Bold values indicates *p* < 0.05 using linear regression.

IL-6, interleukin-6; IL-8, interleukin-8; TNF, tumor necrosis factor; NCI, non-cognitive impairment; CIND, cognitive impairment no dementia; max n, maximum number; MD, mean difference; CI, confidence interval.

^a^Log-transformed.

^b^Adjusted for age, hypertension, hyperlipidemia, diabetes and cardiovascular disease.

## Discussion

The present study found higher plasma osteopontin (OPN) in both vascular cognitive impairment as well as AD without significant CeVD burden. Notably, the increase in OPN levels were significantly associated with MRI markers of CeVD, namely cortical infarcts, lacunes and WMH. This is in line with a previous study which showed positive correlations between OPN and ischemic lesion volume in acute ischemic stroke patients^53^. Several animal stroke models also showed an increase in the mRNA levels of OPN, along with other inflammatory cytokines, suggesting that OPN may be involved in post-CeVD neuroinflammatory responses^25,26^. A recent study has highlighted the potential neuroprotective effect of OPN in cerebral ischemia induced inflammation and oxidative stress^54^, which further supports the hypothesis that the increased OPN reflects a compensatory response towards CeVD-asociated vascular damage and inflammation.

Besides CeVD, OPN may also play a role in AD, as significant associations between higher OPN levels and AD in the absence of CeVD was observed. This is further supported by the correlation between increased OPN levels and brain atrophy observed in our study, suggesting that OPN elevation may be a response to neurodegeneration. This is in line with previous studies which implicated OPN in the clearance of pathogenic beta-amyloid (Aβ) proteins in AD^55^. Given that no significant association was observed between OPN and CIND without CeVD, our results suggest that the compensatory response towards OPN upregulation may only be triggered in later stages of AD and not in pre-dementia. Furthermore, the differing trends observed between CIND with and without CeVD imply that OPN may in involved in different stages of AD vs. VCI; namely, early in VCI but later in AD-associated neurodegeneration. Hence OPN may be a useful biomarker for identifying patients at risk of developing VaD, as previous studies have demonstrated the likelihood of CIND patients with CeVD converting to VaD^56,57^. However, this requires further longitudinal study for validation.

Both neurodegeneration and CeVD are recognized to be the key players in cognitive impairment and dementia. We thus postulated that the atrophy- and CeVD-linked OPN is associated with clinical cognitive performance as well, and our study demonstrated for the first time that increased plasma OPN was associated with poor performance in both global as well as all domain-based cognitive functions, thus expanding upon previous findings of OPN correlations with MMSE^33,34^. The observed associations of OPN with all seven domain-based cognitive functions highlights the potential involvement of OPN, either directly or through regulation of neuroinflammation^27,28^, in the pathophysiology of cognitive impairment. This postulate is further supported by the data showing plasma OPN associations with elevation of peripheral inflammatory cytokines including IL-6, IL-8 and TNF. Our group has also previously reported associations between IL-8 and markers of CeVD, specifically WMH^36^. This in turn implies that neuroinflammation may underlie the link between OPN upregulation and small vessel disease in cognitive impairment and dementia.

Our study has several limitations. Firstly, as the analysis is cross-sectional, the temporal association between OPN and the progression of cognitive impairment is not assessed. Secondly, as cases and the majority of the controls were recruited from memory clinics, who may have had a higher burden of vascular conditions or CeVD, our findings may be less generalizable to the elderly population at large. Thirdly, different processing protocol was used for the measurement of OPN (which was measured in EDTA plasma) and other inflammatory cytokines (which were measurement in SST serum). Although blood was collected into both EDTA and SST tubes from each participant concurrently, caution needs to be taken while interpreting the data as pre-analytic blood processing procedures may affect the expression of biomarkers in the blood^58,59^. Lastly, the precise mechanisms underlying the involvement of OPN in VCI and AD remain unclear. The purported neuroprotective and Aβ-clearing roles of OPN are contrasted with its function in up-regulating proinflammatory cytokines. Indeed, elevated OPN may, depending on stage and type of disease, have both beneficial and detrimental roles, the latter perhaps via maladaptive, chronic upregulation of neuroinflammation leading to tissue injury. However, more studies are needed to examine these postulated mechanisms. Nevertheless, our study’s strengths include the use of 3 T-MRI to grade and classify individuals with CeVD and brain atrophy, and the use of comprehensive neuropsychological assessments to diagnose cognitive impairment and dementia as well as to examine the individual cognitive domains. Moreover, possible confounding effects of demographic characteristics and vascular risk factors were taken into account in our multivariate regression analyses.

## Conclusions

In conclusion, our findings suggest that plasma OPN is a marker for both VCI-associated CeVD as well as AD-associated brain atrophy. The current study also demonstrated OPN’s positive correlation with inflammatory cytokines, as well as negative correlations with neurocognitive performance, further supporting the involvement of inflammation in VCI and AD. However, further longitudinal analyses as well as mechanistic studies are required to elucidate the precise role of OPN in disease pathogenesis and progression as well as its clinical utility as a biomarker or therapeutic target.

## Supplementary Information

Supplementary Information.

## Data Availability

The datasets used and/or analysed during the current study are available from the corresponding author on reasonable request.

## Abbreviations

AD: Alzheimer’s disease
ANOVA: Analyses of variance
APOE: Apolipoprotein E
ARWMC: Age-related white matter changes scale
AUC: Area under curve
CeVD: Cerebrovascular disease
CI: Confidence interval
CIND: Cognitive impairment no dementia
CSF: Cerebrospinal fluid
DSM-IV: Diagnostic and statistical manual of mental disorders, 4th edition
DSRB: Domain-Specific Review Board
EDTA: Ethylenediaminetetraacetic acid
FLAIR: Fluid attenuated inversion recovery
IL: Interleukin
IQR: Interquartile range
MD: Mean differences
MFI: Median fluorescent intensity
MMSE: Mini-Mental State Examination
MoCA: Montreal Cognitive Assessment
MPRAGE: Magnetization prepared rapid gradient recalled echo
MRI: Magnetic resonance imaging
NCI: No cognitive impairment
NINCDS-ADRDA: National Institute of Neurological and Communicative Disorders and Stroke and the Alzheimer's Disease and Related Disorders Association
NINDS-AIRENS: National Institute of Neurological Disorders and Stroke-Association Internationale pour la Recherché et l' Enseignement en Neuroscience
OPN: Osteopontin
OR: Odds ratio
ROC: Receiver operating characteristic
RR: Risk ratios
SD: Standard deviation
SPP1: Secreted phosphoprotein 1
TNF: Tumor necrosis factor
VaD: Vascular dementia
VCI: Vascular cognitive impairment
WAIS-R: Wechsler Adult Intelligence Scale-revised
WMH: White matter hyperintensities
WMS-R: Wechsler Memory Scale-revised
