# Supplementary material for: Plasma osteopontin as a biomarker of Alzheimer’s disease and vascular cognitive impairment
Source: Sci Rep. 2021 Feb 17;11:4010. doi: 10.1038/s41598-021-83601-6 (PMC7889621; doi:10.1038/s41598-021-83601-6)
Supplement: Supplementary file 1 — Supplementary Information. [file 41598_2021_83601_MOESM1_ESM.pdf]

Plasma osteopontin as a biomarker of Alzheimer's disease and vascular cognitive impairment

Yuek Ling Chai, Joyce R. Chong, Ainiah R. Raquib, Xin Xu, Saima Hilal, Narayanaswamy Venketasubramanian, Boon Yeow Tan, Alan P. Kumar, Gautam Sethi, Christopher P. Chen, Mitchell K.P. Lai

Supplementary Information

Chai et al. Supplementary Table 1: Summary of Neuropsychological Battery and Component Tests

Cognitive Domain	Component Test(s)
i. Executive Function:	Frontal Assessment Battery [1]
ii. Attention:	Digit Span, Visual Memory Span [2] and Auditory Detection [3]
iii. Language:	Language: Modified Boston Naming Test [4] and Verbal Fluency [5]
iv. Visuomotor Speed:	Symbol Digit Modality Test [6], Maze Task [7] and Digit Cancellation [8]
v. Visuoconstruction:	Weschler Memory Scale – Revised (WMS-R) Visual Reproduction Copy task [2], Clock Drawing [9] and Weschler Adult Intelligence Scale – Revised (WAIS-R) subtest of Block Design [10]
vi. Visual Memory:	Picture Recall & Recognition Tasks, and WMS-R Visual Reproduction Recall & Recognition Task [2]
vii. Verbal Memory:	Word List Recall & Recognition Tasks [11] and Story Recall Task

References

1. Dubois B, Slachevsky A, Litvan I, Pillon B: **The FAB: a Frontal Assessment Battery at bedside.** *Neurology* 2000, **55:**1621-1626.
2. Wechsler D: **Wechsler Memory Scale-Revised**. 3rd ed. San Antonio, TX: Jovanovich; 1997.
3. Lewis RF, Rennick PM: **Manual for the Repeatable Cognitive Percpetual-Motor Battery**. Clinton Township, MI: Axon; 1979.
4. Mack WJ, Freed DM, Williams BW, Henderson VW: **Boston Naming Test: shortened versions for use in Alzheimer's disease.** *Journal of Gerontology* 1992, **47:**P154-158.
5. Isaacs B, Kennie AT: **The Set test as an aid to the detection of dementia in old people.** *The British Journal of Psychiatry: The Journal of Mental Science* 1973, **123:**467-470.
6. Smith A: **Symbol Digit Modalities Test**. Los Angeles, CA: WPS Inc.; 1973.
7. Porteus SD: **The Maze Test and Clinical Psychology**. Palo Alto, CA: Pacific Books; 1959.
8. Diller L, Ben-Yishay Y, Gerstman LJ: **Studies in Cognition and Rehabilitation in Hemiplegia**. New York: New York University Medical Center Institute of Rehabilitation Medicine; 1974.
9. Sunderland T, Hill JL, Mellow AM, Lawlor BA, Gundersheimer J, Newhouse PA, Grafman JH: **Clock drawing in Alzheimer's disease. A novel measure of dementia severity.** *Journal of the American Geriatrics Society* 1989, **37:**725-729.
10. Wechsler D: **Wechsler Adult Intelligence Scale-Revised**. San Antonio, TX: Harcourt Brace Jovanovich; 1981.
11. Sahadevan S, Tan NJ, Tan T, Tan S: **Cognitive testing of elderly Chinese people in Singapore: influence of education and age on normative scores.** *Age and Ageing* 1997, **26:**481-486.

Chai et al. Supplementary Table 2: ROC analysis of plasma OPN for discriminating each diagnostic outcome from NCI (n=378)

Model	Diagnostic outcome	AUC (95% CI)	Sensitivity (95% CI)	Specificity (95% CI)
**Unadjusted**	CIND without CeVD	0.50 (0.41 – 0.59)	26.6 (8.9 – 97.5)	87.5 (11.3 – 98.8)
	CIND with CeVD	0.63 (0.54 – 0.71)	70.9 (30.4 – 88.6)	58.8 (35.0 – 92.5)
	AD without CeVD	0.80 (0.71 – 0.88)	72.5 (55.0 – 92.5)	85.0 (60.0 – 93.8)
	AD with CeVD	0.71 (0.63 – 0.80)	58.0 (39.1 – 87.0)	82.5 (48.8 – 95.0)
	VaD	0.73 (0.62 – 0.84)	64.5 (41.9 – 96.8)	83.8 (41.3 – 95.0)
**Adjusted***	CIND without CeVD	0.68 (0.59 – 0.76)	59.5 (36.7 – 79.8)	76.3 (53.8 – 92.5)
	CIND with CeVD	0.77 (0.70 – 0.84)	68.4 (53.2 – 93.7)	77.5 (46.3 – 90.0)
	AD without CeVD	0.92 (0.86 – 0.97)	85.0 (72.5 – 97.5)	91.3 (76.3 – 97.5)
	AD with CeVD	0.89 (0.84 – 0.95)	82.6 (71.0 – 92.8)	87.5 (78.7 – 96.3)
	VaD	0.91 (0.85 – 0.96)	90.3 (71.0 – 100)	81.3 (62.5 – 96.3)

ROC = Receiver operating characteristic, OPN = osteopontin, NCI = no cognitive impairment, CeVD = cerebrovascular disease, CIND = cognitive impairment no dementia, AD = Alzheimer's disease, VaD = vascular dementia, AUC = area under curve, CI = confidence interval
*Adjusted for age, education, APOE4 carrier status, hypertension, diabetes and cardiovascular disease.

Chai et al. Supplementary Table 3: ROC analysis of plasma OPN for discriminating each diagnostic outcome from all other diagnoses (n=378)

Model	Diagnostic outcome	AUC (95% CI)	Sensitivity (95% CI)	Specificity (95% CI)
**Unadjusted**	CIND without CeVD	0.51 (0.42 – 0.60)	29.1 (11.4 – 97.5)	86.3 (11.3 – 97.5)
	CIND with CeVD	0.63 (0.55 – 0.72)	67.9 (30.9 – 87.7)	61.3 (37.5 – 92.5)
	AD without CeVD	0.78 (0.69 – 0.86)	68.2 (50.0 – 93.2)	83.8 (53.8 – 93.8)
	AD with CeVD	0.71 (0.63 – 0.80)	58.0 (39.1 – 88.4)	82.5 (46.2 – 95.0)
	VaD	0.73 (0.62 – 0.84)	64.5 (41.9 – 96.8)	83.8 (40.0 – 95.0)
**Adjusted***	CIND without CeVD	0.71 (0.63 – 0.79)	62.0 (38.0 – 84.8)	76.3 (51.3 – 93.8)
	CIND with CeVD	0.76 (0.68 – 0.83)	75.3 (55.6 – 95.1)	68.8 (42.5 – 86.3)
	AD without CeVD	0.92 (0.87 – 0.97)	88.6 (75.0 – 97.7)	87.5 (74.8 – 96.3)
	AD with CeVD	0.88 (0.82 – 0.94)	79.7 (63.8 – 92.8)	86.3 (71.3 – 96.3)
	VaD	0.91 (0.86 – 0.96)	93.6 (67.7 – 100)	77.5 (60.0 – 100)

ROC = Receiver operating characteristic, OPN = osteopontin, NCI = no cognitive impairment, CeVD = cerebrovascular disease, CIND = cognitive impairment no dementia, AD = Alzheimer's disease, VaD = vascular dementia, AUC = area under curve, CI = confidence interval

*Adjusted for age, education, APOE4 carrier status, hypertension, diabetes and cardiovascular disease.
